# Optimization Strategy of Expression Vectors and Regulatory Elements for Enhanced Protein Production in *Bacillus subtilis*

**DOI:** 10.3390/ijms262210812

**Published:** 2025-11-07

**Authors:** Ziru Ye, Puyue Zhang, Zhong Tian, Yong Huang

**Affiliations:** 1 School of Pharmacy, Chengdu University of Traditional Chinese Medicine, Chengdu 611137, China; yeziru@stu.cdutcm.edu.cn (Z.Y.);; 2 Innovative Institute of Chinese Medicine and Pharmacy, Chengdu University of Traditional Chinese Medicine, Chengdu 611137, China

**Keywords:** *Bacillus subtilis*, expression vector, plasmid stability, regulatory element

## Abstract

As a non-pathogenic, Gram-positive strain, *Bacillus subtilis* is well-known for its efficient protein secretion mechanism and versatile microbial cell factory. However, the present *B. subtilis* expression vectors have drawbacks that prevent their industrial use, such as poor stability, low copy number, and low expression efficiency. In recent years, systematic optimization of expression vectors and elements has emerged as a key strategy for enhancing protein production efficiency. Among these efforts, constructing high-copy, stable vector backbones serves as the foundation for improving heterologous protein expression. Further optimization of critical regulatory elements—including regulatory genes, promoters, ribosome binding sites, signal peptides, and terminators—can significantly increase protein yield and process controllability. This review summarizes recent advances in *B. subtilis* expression systems, focusing on vector design and coordinated optimization of regulatory elements. Additionally, it discusses strategies for constructing efficient and controllable expression vectors, offering theoretical insights and technical guidance for future industrial applications.

## 1. Introduction

*Bacillus subtilis* (*B. subtilis*) is ubiquitous in the animal gastrointestinal tracts of diverse organisms [1,2,3], including fish [4], earthworms [5], sheep [6], and alpacas [7], as well as in terrestrial [8], and aquatic [9] environments. It can generate highly resistant dormant spores to withstand harsh environments [10,11]. *B. subtilis* presents a highly advantageous host for biotechnological applications. Unlike *actinomycetes* [12] and *lactic acid bacteria* [13], it requires only simple culture conditions [12] and exhibits a rapid growth cycle [14]. While it shares these operational advantages with *Escherichia coli* (*E. coli*), *B. subtilis* is distinguished by its lack of pyrogenic lipopolysaccharides [15] and its highly efficient protein secretion system [16,17]. These traits render *B. subtilis* a host strain [18,19] to efficiently express pectinases [20], α-amylase [21], and lipase [22,23]. In recent years, its application value has expanded beyond various enzyme production [24,25,26] to encompass numerous fields [27]. *B. subtilis* enzymes and metabolites are widely used in industrial [28,29], agricultural [30,31], and medical applications [32,33], among others. In the industrial sector, it serves as a model strain for producing various enzymes [27]. In the agricultural sector, it is applied as a new biopesticide to promote plant development and perform biocontrol functions [27,34]. In the medical field, it acts as a probiotic to regulate the balance of intestinal flora [27,35]. In the environmental sector, it is utilized as an innovative bioremediation method to break down hazardous compounds [36,37]. *B. subtilis* demonstrates significant application potential in these fields.

**Figure 1:**
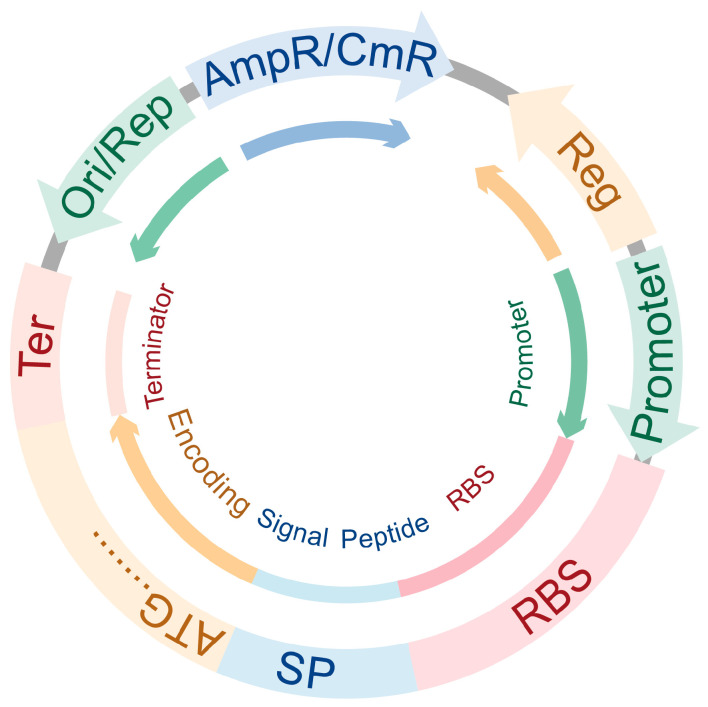
Basic regulatory elements for vectors in *B. subtilis*. Ori/Rep: *E. coli*’/*B. subtilis*’ replicon, indicated by the green arrow; AmpR/CmR: resistance to ampicillin (*E. coli*)/resistance to chloramphenicol (*B. subtilis*), indicated by the blue arrow; Reg: regulatory system genes, indicated by an orange arrow; RBS: ribosome binding sites, indicated by the red line segment; SP: signal peptide, indicated by the blue line segment; Encoding: encoding gene, indicated by the orange line segment; Ter: terminator, indicated by the pink line segment.

However, certain restrictions exist in the large-scale application of *B. subtilis* [38]. The expression vectors have poor stability and a low copy number [38]. Key expression elements exhibit inefficiencies in precise control, transcription, and translation [38]. These issues result in low yields and synthesis challenges of cyclic lipopeptides, linear lipopeptides, and dihydroisocoumarins in *B. subtilis*, thereby restricting their applications in agriculture and medicine [39]. Establishing a stable and efficient expression vector is a prerequisite for the development of *B. subtilis* [40]. Current design strategies focus on constructing efficient and stable vector backbone frameworks [38,40], followed by iterative optimization [38] of key regulatory elements—including regulatory genes, promoters, ribosome binding sites (RBS), signal peptides, and terminators. Additionally, engineering design principles from synthetic biology have been incorporated into vector design via [27] modularization of functional elements, resulting in the development of the ProUSER 2.0 toolbox [41]. Based on the existing research methods, this review systematically sorts out the current optimization strategies for *B. subtilis* expression vectors. The optimization schemes can be divided into two major directions: modify the vector skeleton (comprising *E. coli*/*B. subtilis* resistance genes, *E. coli*/*B. subtilis* replicon, and gene expression elements) and optimize regulatory elements (regulatory genes, promoters, RBS, signal peptides, and terminators) (Figure 1). Inspired by the ProUSER2.0 toolbox design principles, this review further summarizes the above optimization strategies into a systematic optimization framework of “vector skeleton + regulatory systems + expression elements” (Figure 1). This framework is essentially a summary and organization of existing optimization strategies, aiming to provide a clearer analytical perspective for the design of expression vectors. Researchers can employ a “customizable on-demand” strategy to selectively optimize the target functional modules according to distinct expression needs. This classification method not only helps to clarify the internal logic of the existing optimization strategies but also provides an expandable theoretical framework for the rational design of the future *B. subtilis* expression system.

## 2. Optimization Strategies for Vector Stability and Copy Number in *B. subtilis*

Stabilizing the vector skeleton is critical for efficiently expressing foreign proteins in *B. subtilis* [40]. *B. subtilis* expression vectors are categorized into plasmid vectors, integration vectors, and thermosensitive phage DNA (Table 1) [40]. The primary plasmid vectors (e.g., pUB110, pE194), which typically undergo rolling-circle replication, are prone to generating single-stranded DNA intermediates, leading to plasmid instability during cell division [42,43]. Therefore, shuttle vectors are widely employed, allowing recombinant construction in *E. coli*, followed by transformation into *B. subtilis* for expression [42,43,44]. Integrative vectors facilitate stable gene expression via chromosomal integration [45]. However, due to the absence of the replication origin, their copy number is constrained by chromosomal replication [46], limiting expression capacity. Additionally, thermosensitive phage DNA can also serve as an expression vector, though related studies remain limited [47,48]. A key limitation of *B. subtilis* expression vectors is their inherent instability and low copy number (Table 1). To overcome these limitations, optimization strategies are summarized in this section.

### 2.1. Enhancing Vector Stability in B. subtilis

Researchers typically enhance vector stability through three main strategies: integrating expression in *B. subtilis*, engineering plasmid vectors, and modifying host strain genomes (Table 2). (1) Integrated expression in *B. subtilis*. The strategy involved selecting target integration sites and incorporating homologous sequences from the *B. subtilis* chromosome into the *E. coli*–*B. subtilis* shuttle vector [56]. Utilizing the homologous recombination mechanism, the target gene is stably integrated into the *B. subtilis* chromosome for stable expression (pMA5, Table 2). (2) Utilization of BGM and its derivative vectors. The *Bacillus* genome vectors (BGM vectors) refer to derivative strains of *B. subtilis* that harbor integrated pBR322 sequences [55]. Leveraging the natural transformation and homologous recombination mechanisms of *B. subtilis*, the BGM vectors were used to successfully integrate large DNA fragments (>100 kb) into its genome (BGM, Table 2) [57]. However, the conventional BGM vectors exhibit inherent instability in cloned DNA inserts [57]. Due to RecA-dependent transformation, endogenous RecA may cause aberrant recombination between homologous sequences in inserted DNA [57]. To prevent aberrant recombination, one method is to induce RecA expression specifically during genetic manipulations. Consequently, researchers developed a xylose-induced recA-expressing BGM vector (iREX) [57]. Compared to BGM vectors, the iREX vector demonstrated improved DNA stability in *B. subtilis* (iREX, Table 2) [57]. (3) Employing essential genetic modification approaches [58]. Researchers improved plasmid stability by constructing recombinant plasmids carrying the *floB* gene (encoding dihydropterin aldolase) and simultaneously knocking out the endogenous *floB* gene (pl36, Table 2) [58]. This made cellular expression of this essential gene strictly dependent on the presence and maintenance of the plasmid [58]. (4) Screen for the replication origin. The researchers constructed a novel plasmid, pBV03, based on pBV-*ori* and the *E. coli*-*B. subtilis* shuttle vector pUBC01. pBV03 could be stably inherited for 40 generations under non-selective conditions (pUBC01, Table 2) [59]. (5) Modify or knock out strain genes. Genes involved in plasmid segregation and replication were modified or knocked out using the CRISPR/Cas9 system or Cre/loxP technology [60,61]. The *yueB* gene was knocked out in *B. subtilis* 168 (*Bs168*) to construct the *BsΔyueB* strain [61]. The deletion of the phage SPP1 surface receptor gene *yueB* in *BsΔyueB* enhanced plasmid stability (pHT01, Table 2) by minimizing sporulation and improving plasmid segregational stability and host adaptation [61]. (6) Genomic stability optimization. Plasmids are susceptible to structural instability (e.g., mutation, rearrangement, or deletion of plasmid DNA) and segregational instability (leading to plasmid-free cells), thereby introducing genetic heterogeneity into the host population [62]. To address this issue, researchers developed a site-dependent mutation bias (SiteMuB) approach to evaluate the spontaneous mutation rates of identical DNA sequences integrated at different genomic loci, thereby identifying genetically stable sites for foreign gene integration (pHT01, Table 2) [62]. Meanwhile, by deleting error-prone DNA polymerase genes (*yolD*, *yozK*, *yozL*) and enhancing the expression of the nucleotide-excision-repair-related gene *uvrC*, the low-mutation-rate chassis strain ΔDKL (ChassisLMR) was successfully constructed (pHT01, Table 2) [62]. Subsequent deletion of the stress-induced mutation-related transcription factor gene *mfd* not only significantly reduced the mutation rate but also markedly increased neuraminic acid production (pHT01, Table 2) [62]. Furthermore, by combining SiteMuB analysis with the ChassisLMR strain, a substantial enhancement in the genetic stability of the T7 expression system was achieved (pHT01, Table 2) [62]. These strategies have been demonstrated to effectively improve the stability of *B. subtilis* expression vectors.

### 2.2. Increasing Vector Copy Number in B. subtilis

To address the issue of low copy numbers in expression vectors, researchers primarily increase vector copy numbers by modifying replication start sites or augmenting the number of integrated genes (Table 2). (1) Introduce the origin of replication to construct a shuttle vector. The introduction of an *E. coli* origin of replication into the vector pWB980-DB effectively increased its copy number (pWB980, Table 2) [63]. (2) Adjusts the position and orientation of the replication initiation site. The replication initiation site’s position and orientation were systematically optimized, considering the crucial role of the membrane-bound BA3-1 region in plasmid partitioning during cell division [63]. Through this systematic optimization, the high-copy vector pUC980-1 was developed, featuring forward-oriented ori insertion downstream of BA3-1 (pWB980, Table 2) [63]. (3) Mutations modulate key plasmid replication factors, notably RNAI/II and IRIII. Mutations at the RNAI/II and IRIII sites can increase the plasmid copy number (pBR322, pGL3, pCB4170, Table 2) [64,65,66]. (4) Increase the number of expression cassettes within the integrated genes. Based on an independent expression cassette containing the promoter, target gene, terminator, and other elements, multiple independent expression cassette fragments were constructed using molecular biology techniques to increase the integrated gene copy number [53]. The DPEase gene was fused with the P_43_ promoter to form an independent monomeric expression cassette, designated P_43_-DPEase [67]. Using *B. subtilis* 1A751 as the host, a three-copy integrated strain, named 1A751-3DPE, was constructed by sequentially integrating the P_43_-DPEase expression cassette into the *amyE* locus [67]. Despite the increase in copy number and enhanced expression, the 1A751-3DPE strain exhibited significant vector instability, attributable to the tendency of its tandem repeats to undergo circularization (pDG1730, Table 2) [67]. Furthermore, the number of integrated genes can be increased by integrating foreign genes at various sites on the chromosome of *B. subtilis* [53]. Through systematic genome engineering, the P_amyQ_-P_cry3A_-amyQ expression cassette was integrated into six loci (*amyE*, *pksG*, *ppsE*, *cotB*, *ylbP*, and *veg*) of the *B. subtilis* BS2 chromosome (pJOE8999.1, Table 2) [54]. Multi-locus integration concertedly elevated gene dosage and α-amylase productivity [54]. All the aforementioned strategies effectively enhance the copy number of expression vectors in *B. subtilis*.

### 2.3. Current Vectors Optimization Strategies Promises and Challenges

Although various optimization strategies have been developed, methods for improving vector stability and copy number still primarily rely on chromosomal integration in *B. subtilis* and modification of the replication origin [68]. Integration strategy not only circumvents issues such as plasmid loss, metabolic burden, and antibiotic reliance but also mitigates concerns related to production instability, high costs, and ecological unsafety during long-term fermentation [56,58,69]. This high stability has been empirically demonstrated—for instance, an integrated vector derived from the shuttle plasmid pMA5 exhibited increased stability from 72% to 98% [56], underscoring the core advantage of integrated vectors in maintaining genetic stability. However, the low-copy nature of integrated vectors, while beneficial for stability, constrains the expression level of target proteins, hindering high-yield production of enzymes such as xylanase and endoglucanase [70,71]. To augment production, increasing the number of expression cassettes has been attempted to elevate copy number [54]. Yet, due to saturation effects in transcription and translation, copy number and protein yield are not always positively correlated [54]. Excessive insertion of expression cassettes may even compromise vector stability [54]. Moreover, the optimal copy number for maximal expression varies considerably among proteins: for example, β-glucosidase *ganA* and α-amylase require 5 and 6 copies, respectively, whereas protease *aprL* achieves the highest yield with only 1 copy [54]. These differences may be attributed to gene-specific translation initiation efficiency, such as the structural features downstream of the start codon [54]. Although copy number optimization can enhance expression to some extent, integrated vectors generally yield lower protein levels than plasmid vectors [54,56]. For instance, α-amylase production reached 1439.2 U/mL [54] in an integrated vector compared to 4824.2 U/mL [72] in a plasmid vector. Therefore, achieving high expression of heterologous proteins while maintaining the high stability of integrated vectors remains a critical challenge for future research.

Shuttle vectors engineered with the replication origin from *E. coli* generally exhibit high copy numbers and elevated expression levels [47]. However, their expression efficiency is often gene-dependent and lacks broad applicability. For example, the plasmid vector pWB980 yields 6125 U/mL of the alkaline protease AprE [73], but only 0.359 U/mL of xylanase [71]. To improve target protein production, conventional strategies such as promoter optimization and adjustment of expression elements are frequently employed. Nonetheless, these strategies are generally specific to individual proteins and lack broad applicability. Thus, there is a critical need to develop vectors that combine high copy number, high stability, and wide applicability across diverse proteins. The pUC980-1/2 shuttle vectors provide a new approach for constructing such vectors. Constructed by deleting the *ble* gene from pWB980 and incorporating the *E. coli* replication initiation site, this modified vector shows a substantial increase in copy number—from 134 to 450—while maintaining 99–100% stability after 30 days of continuous subculturing [63]. In terms of protein expression, pUC980-1/2 achieved yields of 5200 U/mL for alkaline pectate lyase (PelN), 21,537 U/mL for alkaline protease (Spro1), and 187 U/mL for pullulanase (PulA11) [63]. Notably, the expression levels of PelN and Spro1 surpassed all previously reported values [63,74,75]. Although the pullulanase yield did not exceed the current maximum (138.69 U/mL), it still demonstrates significant potential for enhancement [63,76]. In conclusion, pUC980-1/2 shows great promise as an efficient vector for heterologous protein expression. Although not yet as extensively applied as commonly used plasmids, future research may pursue two main directions: first, direct application of this vector for expressing and optimizing a wider range of heterologous proteins; second, adoption of its engineering strategy to develop novel plasmid systems featuring high copy number, high stability, and broad applicability.

## 3. Optimization Strategies for Regulatory Systems in *B. subtilis*

Efficient and precise expression of target genes remains a central challenge in synthetic biology [77]. The *B. subtilis* commonly employs two types of exogenous expression systems: constitutive and inducible [77]. Constitutive systems lack temporal and dose control, leading to suboptimal resource allocation between cell growth and product synthesis, which ultimately limits yield [77]. To dynamically balance growth and production, inducible systems are widely adopted for precise regulation of both the timing and level of gene expression [77]. Commonly used chemical induction systems in *B. subtilis* rely on inducers such as isopropyl thiogalactoside (IPTG)/lactose [78,79,80,81], xylose [82,83,84], mannitol [85], maltose [86,87], methanol [88], and glycerol [77] (Table 3). Nevertheless, these systems suffer from several limitations, including low inducer utilization efficiency, carbon catabolite repression (CCR), high cost, cytotoxicity, and leaky expression (Table 3). To overcome these challenges, recent research has pursued two main strategies: first, engineering the host genome and developing autoinduction or optogenetic systems to reduce cost and toxicity while improving inducer efficiency; second, constructing dual transcriptional–translational regulatory circuits to suppress leakage. This subsection reviews recent advances and optimization strategies addressing these issues.

### 3.1. Enhancing the Utilization Efficiency of Inducers in B. subtilis

To overcome low inducer utilization, researchers focus on improving inducible transporter efficiency and reducing carbon catabolite repression (CCR). Strategies include overexpressing inducer transporters [90,91] and modifying inducer metabolic pathways [92], both effectively enhancing inducer utilization. Taking the transporter AraE as an example, it exhibits broad substrate specificity and participates in the degradation and transport of arabinose-containing polysaccharides, xylose, and galactose [93]. AraE overexpression [90,91] or engineering of its related metabolic pathways [92] has been shown to substantially improve the utilization efficiency of inducers such as xylose (Table 4). Key optimization strategies include the following: (1) integration of the AraE expression cassette into the *B. subtilis* chromosome to facilitate xylose transport (Table 4) [90,91]. (2) Knockout or mutated genes that negatively regulate transporter proteins [94]. The *araR* gene encodes AraR, a repressor that binds to the *araE* promoter, inhibiting *araE* expression. Disrupting the araR gene reduces this repression (Table 4) [90,93]. (3) Modification of the regulatory pathways of inducer metabolism [92]. Taking the xylose metabolic pathway as an example, a metabolic engineering strategy was implemented to enhance the supply of the Fenycin precursor, pyruvate. This involved overexpressing *yjhG* (encoding xylonate dehydratase) and *yjhH* (encoding 2-keto-3-deoxy-D-xylonate aldolase) to channel carbon flux, coupled with knocking out *ackA* (acetate kinase) and *ldh* (lactate dehydrogenase) to minimize competitive pyruvate consumption. Furthermore, an auxiliary pathway comprising *aldA* (aldehyde dehydrogenase), *aceB* (malate synthase), and *mdh* (malate dehydrogenase) was introduced to redirect the byproduct glycolaldehyde into the TCA cycle. This integrated approach not only improved xylose uptake efficiency but also increased Fenycin yield by 87% (Table 4) [92]. (4) Knockout of sugar hydrolase genes effectively enhances expression performance. Taking the maltose regulation system as an example, deletion of the maltose hydrolase genes *malL* and *yvdK* not only significantly increased the activity of promoter P_malA_ and green fluorescent protein (GFP) expression but also resulted in superior expression of luciferase and D-aminoacylase compared to the constitutive expression system (Table 4) [95]. (5) Reducing the CCR effect enhances the uptake and utilization of inducers such as xylose and maltose [84,96,97]. In *B. subtilis*, the presence of glucose promotes the binding of the CcpA protein complex to *cre* sequences within promoters [98,99], thereby inhibiting transcription initiation. To address this, researchers have employed two main strategies: engineering the *cre* sequence in promoters to reduce the catabolite control protein A (CcpA) binding affinity and mutating the CcpA/HPr kinase (HprK/P) genes [97,100] to attenuate the CCR effect (Table 4). Furthermore, surfactin (encoded by the *srfA* gene) mitigates CcpA-dependent CCR [101]. *srfA* deficiency downregulates xylose/galactose metabolic genes and upregulates CcpA, impairing non-preferred carbon source utilization (Table 4) [101]. Thus, exogenous surfactin or *srfA* engineering may serve as a potential strategy to alleviate CCR and improve protein expression [101].

### 3.2. Implementing Alternative Regulatory Systems to Reduce Inducer Toxicity and Costs

The challenges posed by the high cost and toxicity of chemical inducers [79,103] necessitate a re-evaluation of current regulatory systems. To address these issues, researchers have primarily developed three types of regulatory systems for dynamic pathway control: environment-responsive, metabolite-responsive, and quorum-sensing (QS) systems [104]. (1) Environment-responsive systems utilize external signals like light, temperature, or pH to initiate gene expression [104]. Based on optogenetics, the CcaSR v1.0 system was the first optimized light-controlled gene expression system for *B. subtilis* (Table 4) [102]. Its molecular mechanism involves the sensor kinase CcaS, which, in the presence of the phycocyanobilin (PCB) cofactor, is converted to holo-CcaS under green light [102]. Holo-CcaS then phosphorylates the response regulator CcaR via its histidine kinase activity, activating transcription from the P_cpcG2-172_ promoter [102]. This activation is reversibly inactivated under red light, terminating transcription [102]. This bidirectional switch offers precise spatiotemporal control, holding potential for studying complex processes like sporulation and biofilm formation in *B. subtilis* [102]. (2) Metabolite-responsive systems dynamically regulate gene expression based on the concentration of specific pathway intermediates (e.g., glyceraldehyde-3-phosphate, G3P) [77,104]. While CCR can hinder inducible promoters (e.g., P_xylA_, P_glv_, P_AOX1_), strategic media adjustments can exploit CCR to create auto-induction systems [77]. For instance, in a glycerol system for *B. subtilis*, CCR ensures preferential glucose consumption. Upon glucose depletion, glycerol is metabolized to G3P via GlpF and GlpK. G3P then binds the anti-terminator protein GlpP, disrupting the intrinsic terminator of the P_glpD_ promoter and subsequently initiating recombinant protein expression [77]. Hence, the timing and expression intensity of gene induction can be regulated by adjusting the ratio of glucose to glycerol [77]. Moreover, the activity of this system is higher than that of the strong constitutive promoter P_43_ [77]. (3) Quorum-sensing (QS) systems enable auto-induction at high cell density, achieving a “grow first, produce later” strategy [104]. Examples in *B. subtilis* include ComQXPA [105,106], LuxI/LuxR [107,108], and DSI-AIPDS [109]. The ComQXPA quorum-sensing system activates Psrf promoter transcription through phosphorylated ComA (ComA~P) accumulation, establishing an auto-inducible expression mechanism [105,106,110]. It yielded 80.2 U/mL of pullulanase in *B. subtilis*, representing 0.36 times the yield of the most effective constitutive promoter, P_566_ [105]. The LuxI/LuxR system originated in the *Vibrio fischeri*. This system can generate Luxr-AHL complexes with the increase in N-acylhomoserine lactones (AHLs) content [111]. This complex can specifically recognize and bind to the *lux-box* sequence in the promoter region, thereby activating the transcription of downstream genes and facilitating the auto-induced expression mechanism [107,108,111]. Notably, the system has demonstrated expanded utility for industrial enzyme production [111]. Experimental studies have verified that coupling the LuxI/LuxR system with modifying promoter methods can enhance extracellular amylase yields by 2.7–3.1-fold compared to conventional P_veg_ promoter controls [111]. These results underscore the significant potential of the LuxI/LuxR system in microbial metabolic engineering applications [111]. Furthermore, the researchers successfully developed a dual-signal input auto-induced protein degradation system (DSI-AIPDS) by integrating a LuxI/LuxR system, an induced ssrA/SspB degradation system, and an AND-gate logic circuit [109]. The successful construction of the DSI-AIPDS system not only addressed key issues related to *B. subtilis*, such as cell lysis, one-time activation, and damage to new cells, but also significantly enhanced the expression level of the target protein [109].

### 3.3. Employing a Dual Transcription-Translation Strategy to Minimize Leaky Expression

**Figure 2:**
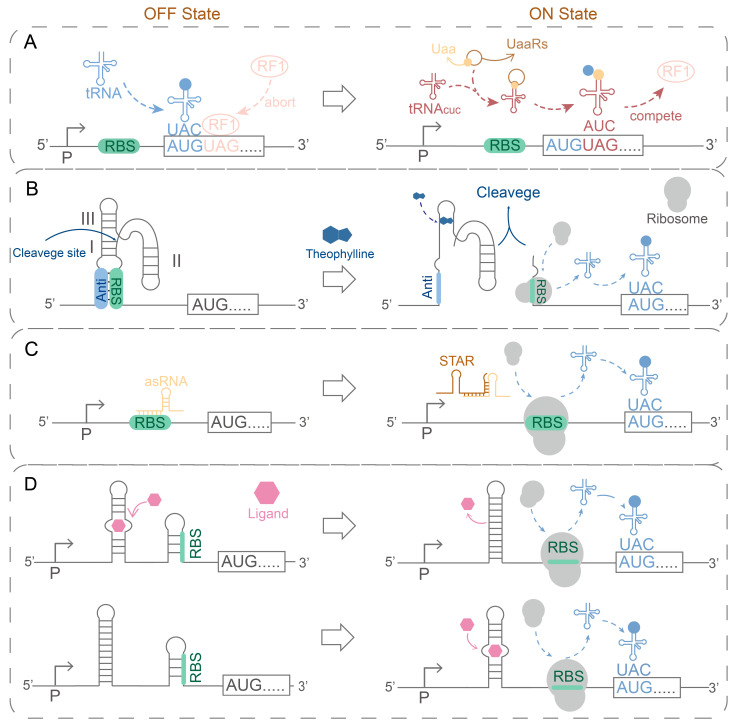
Commonly used translation regulatory systems. (**A**) Uaa system: Under the action of encoding UAA-specific aminoacyl tRNA synthetase (UaaRS) and homologous tRNAcuc, Uaa and releasing factor (RF1) competitive recognition bind the UAG sequence to enable translation. This process is indicated by the red arrow in figure (**A**). The blue arrow indicates that tRNA transports amino acids to ribosomes. The pink arrows indicate the RF1 binding to the UAG sequence. (**B**) Theophylline ribozyme: Theophylline ribozyme cleaves the ribosome binding site (RBS) complementary sequence in the presence of theophylline, releasing the RBS sequence and initiating translation. This process is indicated by the light blue arrow in figure (**B**). (**C**) Antisense RNA: The STAR binding to asRNA obstructs the RBS sequence to form a termination hairpin. This process exposes RBS and starts translation. This process is indicated by the light blue arrow in figure (**C**). (**D**) Riboswitches: The RBS is masked or exposed by changes in the secondary structure of the riboswitches. This dynamic control makes ribosome binding or separation of RBS at ligand concentrations, thereby exerting precise control over translation. This process is indicated by the light blue arrow in figure (**D**). References for details [112,113,114,115].

Introducing a dual transcription-translation regulatory system can effectively mitigate leakage expression [112]. This system integrates regulatory elements into the translation step, enabling “zero expression” during host growth through combined transcriptional and translational control [112]. Current design methods (Figure 2) encompass the unnatural amino acid (Uaa) system (Uaa system) [113,114], ribozyme [115], antisense RNA (asRNA) [112], and riboswitch [115]. (1) Uaa system (Figure 2A): The Uaa (noncanonical amino acid, ncAA) system includes an amber codon, Uaa-specific aminoacyl-tRNA synthetase (UaaRS), and cognate suppressor tRNA (tRNA_CUA_) [112,114,116,117]. Translation regulation is achieved by inserting an amber codon (UAG) to interrupt translation [113,114]. Upon external Uaa addition, it is imported into the cytosol, recognized by UaaRS, and charged onto tRNA_CUA_ [113,114]. This suppresses the amber codon and enables translation initiation [113,114]. Currently, the primary Uaa used for translation regulation is 3-iodo-L-tyrosine and O-methyl-L-tyrosine [114,117]. So far, the Uaa system has seen limited use in *B. subtilis*, primarily in the BacAmp system [117]. Researchers introduced the O-methyl-L-tyrosine-mediated Uaa system into *B. subtilis* and constructed the BacAmp system, which integrates the LacI repressor and an O-methyl-L-tyrosine-responsive expression module [117]. This system not only enables dual transcriptional and translational control of the homologous recombination gene recA but also maintains the homologous recombination probability below 10^−9^ [117]. This result highlights the potential of the Uaa system in mitigating leakage expression in *B. subtilis* [117]. (2) Ribozyme (Figure 2B): Insertion of a ribozyme gene upstream of the translation initiation site enables ligand-dependent self-cleavage, leading to RBS exposure and translation initiation [112,115]. This strategy has been shown to reduce leakage [118] and improve protein expression in *B. subtilis* [119]. (3) Antisense RNA (Figure 2C): Antisense RNA is a single-stranded RNA. It regulates exogenous gene expression by binding to specific mRNA through complementary sequences, thereby inhibiting mRNA translation [112]. Currently, the dual regulation of antisense RNA on transcription and translation has achieved a leaky expression level of less than 0.1% and a gain of up to 923-fold [120]. (4) Riboswitches (Figure 2D): Riboswitches are regulatory elements located in the 5′-untranslated region (5′-UTR) that control the ON/OFF state of downstream genes [115]. Typically inserted upstream of the target gene within the 5′-UTR [115], riboswitches in the OFF state block the RBS through the formation of an inhibitory stem [112,116]. In the ON state, ligand binding to the aptamer domain induces conformational changes that expose the RBS and initiate translation [112,116]. Research has shown that riboswitches exhibit low leakage and high expression levels in *B. subtilis* [118,121]. In addition, mutation of repressor protein expression genes effectively suppresses leakage expression. For example, site-directed mutagenesis of *lacI* resulted in a tenfold reduction in leakage [122]. Multiple strategies effectively reduce leakage in *B. subtilis*.

### 3.4. Current Regulatory Systems Promises and Challenges

In industrial production, the temporal regulation of product expression in microbial strains is critical: premature expression increases metabolic burden, while delayed expression compromises maximum yield [104]. To precisely balance bacterial growth and production for optimal benefits, researchers have developed regulatory systems in *B. subtilis*. Currently, inducible systems such as IPTG/lactose, xylose, sucrose, and maltose-based regulators (Table 3) are commonly employed. The IPTG/lactose system, renowned for its high induction efficiency and broad applicability, has been widely utilized in novel system development [117] and protein expression [117,123,124]. For instance, the BacAmp system has been successfully constructed based on the IPTG/lactose system [117]. The IPTG/lactose system efficiently expresses N-acetylneuraminic acid [117], 2′-Fucosyllactose [123], and β-galactosidase [124], yielding 6.3 g/L [117], 9.67 g/L [123], and 14 × 10^4^ units [124], respectively. However, this system is characterized by several drawbacks, including leaky expression, the toxicity of the inducer IPTG, and high costs. While the dual regulatory mechanism can effectively mitigate the issue of leaky expression, its application in industrial contexts not only elevates production costs but also necessitates the additional step of IPTG removal during subsequent product purification, complicating the purification process. In contrast, xylose/maltose systems offer advantages such as cost-effectiveness, availability, safety, and non-toxicity. These systems have facilitated the production of fengycin (376.58 mg/L) [90], D-tagatose (39.57 g/L) [125], and maltotetraose-forming amylase (3.9 mg/mL) [126]. Nevertheless, they exhibit limitations like low inducer utilization and susceptibility to carbon catabolite repression (CCR) [68]. The sucrose-regulated system employs a low-cost, non-toxic inducer; however, the promoter is characterized by low strength and a tendency for leaky expression [68]. Although researchers have effectively addressed the issues of low inducer utilization, the CCR effect, and leaky expression through their optimization strategies, these regulatory systems continue to encounter challenges related to high-cost inducers, cell growth cycle monitoring, and the operation of adding inducers in industrial applications. These limitations have spurred the development of light-controlled and auto-induction systems. The engineered CcaSR v1.0 optogenetic tool, the first of its kind in *B. subtilis*, exhibits a high dynamic range (>70-fold) and sensitivity to low light intensity [102]. However, it requires higher light intensity for activation and shows slower response kinetics compared to *E. coli*, highlighting both the potential and unique challenges of adapting complex optogenetic tools to this host [102]. Auto-induction systems eliminate the need for external inducers by responding to cell density, yet limited studies have been conducted, and expression efficiency remains suboptimal. For example, pullulanase production using auto-induction reached only 80.2 U/mL, insufficient for industrial demands [105,106]. Despite current limitations, these emerging systems not only provide new tools for regulation in *B. subtilis* but also offer insights for transitioning inducible systems to auto-induction frameworks.

## 4. Optimization Strategies for Expression Elements in *B. subtilis*

The rational design of expression elements, including promoters, RBS, signal peptides, and terminators, is crucial for efficient heterologous protein expression in *B. subtilis*. A strong promoter ensures high-level transcription initiation. Rational RBS design maximizes translation initiation efficiency. Employing an appropriate signal peptide facilitates efficient protein secretion and supports proper folding and stability. Furthermore, efficient terminators prevent transcriptional readthrough, enhance plasmid stability, and extend mRNA half-life. Consequently, systematic optimization of these components is crucial for developing high-performance and controllable *B. subtilis* expression systems.

### 4.1. Enhancement of Transcriptional Level via Promoters in B. subtilis

A promoter (P) is an essential DNA sequence located upstream of a target gene, required for the precise initiation of transcription [127]. The core promoter region is typically defined as spanning from 200 bp upstream to 100 bp downstream of the transcription start site (TSS) [68]. A typical prokaryotic promoter contains conserved regions, including the TSS, the −35 motif (TTGACA), and the −10 motif (TATAAT) [68]. In *B. subtilis* expression vectors, commonly used promoters are categorized as either constitutive or inducible [128]. Constitutive promoters mediate continuous gene expression in the absence of an inducer [128], while inducible promoters regulate expression in response to environmental factors or chemical inducers [68]. However, the low efficiency and inadequate expression levels of both constitutive and inducible promoters constrain their application in *B. subtilis*, thereby limiting the efficient production of heterologous proteins [68]. Thus, developing effective promoter engineering strategies is imperative. These strategies primarily encompass three approaches (Table 5) [127]: screening strong promoters, modifying promoter sequences, and constructing dual promoters. (1) Screening strong promoters. The selection of a stronger promoter improves the transcriptional level of the target gene and consequently boosts protein expression. For example, when compared to P_43_, a constitutive promoter extensively used in *B. subtilis*, P_spoVG_ and P_yvyD_ demonstrate a significant advantage by exhibiting higher transcriptional levels and superior protein expression (Table 5) [129,130]. (2) Truncating the upstream element or increasing its adenine-thymine (AT) content can effectively enhance transcription efficiency (Table 5) [129]. (3) Mutating the −10 and −35 motifs. For promoters containing non-standard −10 and −35 motifs, site-directed mutagenesis towards the conserved sequences (TATAAT and TTGACA) serves as an effective strategy for enhancement [78,131]. A notable demonstration is the optimization of P_grac01_ to P_grac100_. This was achieved by increasing the upstream AT content, mutating the −35 motif (TTGAAA→TTGACA), and altering the −15 motif (TCT→ATG). Consequently, in *B. subtilis*, P_grac100_ exhibited a 27-fold increase in GFP expression and a 9.2-fold increase in β-galactosidase activity compared to P_grac01_ [132]. This conventional approach strengthens transcription by optimizing key promoter recognition elements. In contrast, certain native promoters employ more sophisticated regulatory mechanisms. A notable example is the MgsR-dependent promoter P_ydbD_, wherein the native −35-like motif acts as an inhibitory sequence. This element induces RNA polymerase stalling to repress transcription initiation. Full promoter activation is only achieved when MgsR binds to its upstream regulatory site; this binding repositions the polymerase to the canonical −35 and −10 motifs, thereby relieving the repression [133]. (4) Optimizing the spacer sequence length between the −35 and −10 regions. The length of the spacing between the −35 and −10 motifs determines the spatial conformation of RNA polymerase [107]. An excessively large or small spacing affects the geometry of RNA polymerase, thereby influencing promoter strength to varying extents [107]. By precisely controlling the spacing and selecting the optimal 17 bp spacing sequence, the efficiency of the promoter can be maximized [68]. (5) Construction of dual promoters. While most native promoters function as a single copy to initiate transcription, this often fails to achieve high-level expression [127]. To create stronger promoters, researchers have developed tandem dual-promoter systems, which increase the copy number of core promoter sequences to enhance gene expression [127]. Studies demonstrate that dual promoters elevate both transcriptional activity and recombinant protein yield compared to single promoters (Table 5) [72,130,134,135]. (6) Construction of hybrid promoters. This strategy employs the fusion and assembly of two or more distinct promoter components to construct a novel promoter, thereby enhancing transcriptional efficiency or conferring novel regulatory properties [127,136]. For example, inserting gamO_2_, the binding site of the transcription factor GamR located in the P_gamA_ promoter, into the P_veg_ promoter of *B. subtilis*, thereby constructing synthetic promoters P_vg1_ and P_vg3_. They cannot only respond to intracellular glucosamine-6-phosphate (GlcN6P) but also exhibit greater strength than P_veg_. These optimization strategies effectively overcome the efficiency limitations of *B. subtilis* promoters, significantly enhancing heterologous protein production.

### 4.2. Augmentation of Translational Rate Through RBS in B. subtilis

The ribosome binding site (RBS) is a cis-acting element in prokaryotic mRNA, located upstream of the start codon (AUG). The RBS sequence is composed of the Shine–Dalgarno (SD) sequence, the start codon, and the short-interval sequence in between [138]. The SD sequence facilitates translation initiation by base-pairing with the anti-SD sequence at the 3′ terminus of the 16S rRNA, thereby promoting the recruitment of the 30S ribosomal subunit to the initiation site [138]. The RBS sequence is a key determinant of translational efficiency and, ultimately, recombinant protein yield, making its optimization a central strategy in synthetic biology for enhancing gene expression. Given its significance to the translation initiation rate, RBS has been extensively studied and optimized. Currently, there are several methods for optimizing RBS sequences (Table 5): (1) screen the optimal RBS sequence. This is typically achieved through two predominant strategies: firstly, the selection of strong RBS sequences derived from endogenously or heterologously highly expressed genes in model organisms such as *B. subtilis* or *E. coli* [130]; secondly, the design, optimization, and prediction of RBS sequences with high translation initiation efficiency using the computational tools like RBS Calculator v2.0 (https://salislab.net/software/, accessed on 2 November 2025) [130], RBS Calculator (https://salislab.net/software/, accessed on 2 November 2025) [72,137]. (2) Selection of a strong SD sequence. Studies have revealed that in *B. subtilis*, the canonical SD sequence 5′-UAAGGAGG-3′ (designated as a “strong” SD sequence) exhibits maximum complementarity to the anti-SD sequence of 16S rRNA (5′-ACCUCCUUA-3′). Substituting the original RBS with one containing this strong SD sequence not only significantly enhances mRNA stability but also markedly increases the translation initiation rate (by 93-fold) and protein expression (Table 5) [138]. (3) Modifying the short-interval sequence length between the SD sequence and the start codon. Studies have determined that the optimal spacing between the RBS and the start codon in *B. subtilis* typically ranges from 7 to 9 nucleotides (nt) [138,145]. Consequently, protein expressions can be effectively enhanced by precisely adjusting the interval length to fall within this optimal range [139,143,146]. (4) Computational prediction and saturation mutagenesis of RBS sequences. Identification of regions most critical to translation initiation efficiency was achieved using the RBS Library Calculator, with subsequent saturation mutagenesis of these locations proving to be an effective strategy for protein expression enhancement (Table 5) [140,143,147]. (5) Inserting an mRNA leader upstream of the target gene translation sequence [148]. This sequence is composed of multiple concatenated tandem repeats of the RBS sequence, forming mRNA sequences with multiple RBS [148]. It was demonstrated that when the mRNA leader sequence incorporated six RBS, the fluorescence intensity of GFP was 5-fold greater than that of the mRNA leader sequence containing only one RBS [148]. This design enables protein translation from multiple sites, thereby effectively boosting protein translation efficiency [148]. Specifically, the aforementioned strategies effectively boost RBS-mediated translation rates.

### 4.3. Improvement of Secretion Efficiency by Signal Peptides in B. subtilis

Signal peptide (SP) is a type of tag employed to localize membrane, secretory, and lysosomal proteins [38]. It is situated at the N-terminal or C-terminal of proteins and consists of 15 to 30 amino acid residues [38]. In *B. subtilis*, SPs direct proteins to the extracellular environment in the late phase of secretion and are cleaved off by signal peptidases to achieve protein release. Their canonical structure consists of the N-terminal, H-terminal, and C-terminal. SPs significantly influence protein secretion efficiency and production levels, making their rational design and optimization a key approach in synthetic biology for improving recombinant expression [140]. Contemporary optimization strategies center on three aspects (Table 5): (1) Screening for a signal peptide compatible with the target protein. The efficiency of protein secretion is co-determined by the synergistic effect of the signal peptide and the target protein [140]. Studies have shown that the same signal peptide may exhibit significant efficacy variations when mediating the secretion of different proteins [141,142]. Therefore, although signal peptides such as SP_aprE_, SP_pel_, and SP_yoaW_ are commonly used in heterologous expressions and are generally efficient, they may not be optimal for other proteins expressed in *B. subtilis* [140]. Based on this, signal peptide screening has become the most effective and commonly used strategy to enhance protein secretion efficiency in *B. subtilis* (Table 5) [26,143]. (2) Overexpressing signal peptidases or employing tandem signal peptides enhances cleavage and secretion efficiency [149]. The tandem signaling peptide P_43_-SP_amyQ_-SP_BsGGT_ enhanced the extracellular expression activity of γ-glutamyl transpeptidase by 63.43% relative to the single signaling peptide P_43_-SP_amyQ_ [149]. Moreover, the overexpression of signal peptidases SipS, SipU, and SipW elevated the extracellular expression activity of γ-glutamyl transpeptidase by 12.82%, 3.78%, and 5.83%, respectively [149]. (3) Modifying the conserved structural regions of signal peptides. *B. subtilis* signal recognition particles show a preference for highly hydrophobic signal peptides. Based on this principle, modifications can be made to the number of positively charged amino acids at the N-terminal and the hydrophobicity strength at the H-terminal [150]. There are three methods for modifying the N-terminal region of a signal peptide: mutating the original amino acid to a basic amino acid (K/R) [151,152], directly inserting a basic amino acid (K/R) at the N-terminal [151,152], and performing saturation mutation on the original amino acid [153]. Through the augmentation of basic amino acid residues in the N-terminal region, the enzymatic activities of amylase, methyl parathion hydrolase, and nattokinase were enhanced by 1.7-fold, 6.6-fold, and 5.2-fold, respectively [153]. Furthermore, enhancing the hydrophobicity of the H-terminal effectively increased the efficiency of protein secretion [153]. Researchers have mutated polar amino acids at the H-terminal to non-polar amino acids to enhance hydrophobicity and improve extracellular enzymatic activity [153]. For example, the application of SP_Bgamy_, characterized by an H-terminal hydrophobicity of 72.7%, increased alkaline protease activity expression by approximately 1000 U/mL, in comparison to SP_ypr_, which has an H-terminal hydrophobicity of 64.5% [150]. (4) Add the superfolder green fluorescent protein (sfGFP) tag to the N-terminal protein. The strategy of using specific proteins (e.g., type I L-asparaginase or sfGFP) to guide the secretion of target proteins has been validated in *B. subtilis* without the involvement of a traditional signal peptide [154]. In particular, sfGFP has been employed for N-terminal fusion due to its capacity to mediate heterologous protein secretion, high translation efficiency, and rapid folding rate, leading to increased protein yield [154]. Experimental results confirmed that sfGFP enhanced glutaminase activity from 11.9 U/mL to 26 U/mL, surpassing the performance of traditional signal peptides and indicating its considerable potential for improving heterologous protein expression [154]. (5) Employing fusion tags at the C-terminal or N-terminal of the protein. The strategic fusion of tags to the N- or C-terminal of a target protein is a widely adopted approach in *B. subtilis* expression systems to enhance the yield and solubility of heterologous proteins and to facilitate purification [155]. Commonly used tags range from short peptides, such as polyhistidine (Poly-His), FLAG, and Strep-tag, to larger proteins like maltose-binding protein (MBP) and small ubiquitin-like modifier (SUMO) [155]. These tags operate through distinct mechanisms. Larger tags, such as MBP (~40 kDa), primarily prevent protein aggregation, thereby enhancing solubility [155]. In contrast, smaller tags, such as SUMO (~11 kDa), can act as intrinsic folding templates, promoting the correct folding of the target protein and consequently improving its stability and yield [155,156]. For example, the fusion of a StrepII-SUMO tag increased the expression of *E. coli* alkaline phosphatase (PhoA) in *B. subtilis* by fivefold [156]. The choice of fusion terminus has a critical influence on the expression outcome. As the N-terminal sequence directly impacts the initiation of transcription and translation, C-terminal tagging is generally preferable for highly expressed genes, whereas N-terminal fusion can be inhibitory [155]. Interestingly, this rule is not absolute [155]. Research indicates that for poorly expressed genes like *egfp*, an N-terminal 6xHis tag can boost expression by up to 15-fold, likely by optimizing the codon adaptation index of the first 10 codons [155]. Conversely, this same strategy suppresses the yield of highly expressed genes, such as *gfp+* [155]. Therefore, the application of fusion tags is not universal and needs to be tailored to the specific characteristics of the target protein.

### 4.4. Optimization of Termination Efficiency with Terminators in B. subtilis

A terminator is a DNA sequence signaling transcription termination for RNA polymerase. Typically located downstream of structural genes, it prevents crosstalk between transcription units, recycles RNA polymerase, and prolongs mRNA stability. Loss or impairment of the terminator function disrupts normal transcription termination, resulting in downstream read-through and compromised gene stability [157,158]. Hence, the precise termination of transcription by terminators is crucial for maintaining the accuracy and stability of gene expression. In *B. subtilis*, terminators can be categorized as Rho-dependent or non-Rho-dependent (intrinsic terminators) [159]. Rho-factor-dependent termination necessitates the assistance of the RNA-binding protein Rho [160,161]. Rho terminates transcription through a translocation operation [160,161]. In contrast, Rho-independent relies on its own GC-rich inverted repeat sequence and the subsequent poly-U structure to achieve transcription termination [162,163]. *B. subtilis* predominantly utilizes the Rho-independent termination mechanism. This section focuses on summarizing the optimization strategies for this type of terminator. The terminator optimization strategy encompasses three main aspects. (1) Screening for high-efficiency terminators [164,165,166]. This principle dictates that a higher termination efficiency more significantly upregulates upstream gene expression while effectively suppressing downstream gene expression. For instance, in a study evaluating 10 terminators from *B. subtilis* and 5 from phages, the TB5 terminator demonstrated an efficiency of 98% [144]. This strong terminator (TB5) resulted in a 2.2-fold upregulation of the upstream GFP and a concurrent 28.4-fold downregulation of the downstream mCherry protein (Table 5) [144]. (2) Analyzing the sequence of terminators [167,168], their structure [168,169], the free energy (ΔG) [106] of the stem-loop region, the U-tract, and the polyA/polyU pairing [170], and their correlations with termination efficiency. When the number of U-tracts is fixed, the termination efficiency exhibits a negative correlation with ΔG [170]. The termination efficiencies of terminators characterized by U-tracts within the range of 6–8 nt consistently exceed 80% [170]. Furthermore, the termination efficiency of terminators exhibiting the polyA/polyU pairing demonstrates a negative correlation with ΔG [170]. (3) Mutating the sequence of terminators, designing tandem terminators or artificial terminators, and then verifying their versatility within the host [170]. The researchers discovered that the termination efficiency increased by approximately 22% and 65% when employing weak-weak (TB10-TB10) and weak-strong (TB10-TB5) terminators in tandem, respectively, while the level of GFP expression rose from less than 7.5 × 10^3^ to 1.0 × 10^4^ and 1.5 × 10^4^, respectively [144]. Consequently, applying these strategies can rapidly enhance the performance of the scarce and often weak natural terminators.

### 4.5. Current Expression Elements Optimization Strategies Promises and Challenges

Current strategies for optimizing expression elements generally involve screening the best-suited elements for a target protein, followed by sequential optimization of individual elements (e.g., promoter, signal peptide, terminator), engineering their sequences or structures, and finally constructing tandem elements (e.g., dual promoters, dual signal peptides, dual terminators) [127,144]. Among these, sequential optimization is the most used and effective strategy, which not only enhances element efficiency but also significantly increases the yield of many proteins (Table 5) [127,135,144]. However, this strategy faces several challenges. (1) The selection of expression elements must consider compatibility with the target protein. Studies show that even strong promoters, highly efficient signal peptides, or strong terminators are not necessarily optimal for expressing different proteins [130,140,141,144]. For instance, for β-mannanase expression, the yield with the P_lapS_ promoter was more than 10 times higher than with P_43_, but for BlAase expression, it was only one-fifth of that with P_43_ [141]. Similarly, dual terminators TH1.5b-TB5 and TB10-TB5 (termination efficiency > 90%) enhanced the expression of aspartate ammonia-lyase much more significantly than β-glucuronidase [144]. Therefore, no universal expression element exists that is broadly suitable for the high-level secretory expression of diverse proteins [130,140,141,144]. (2) A mismatch between promoter transcriptional level and translation efficiency can limit protein expression [130,135,141,143]. While employing dual promoters can enhance transcription, it does not always increase protein yield and may sometimes result in a lower yield than using a single promoter [143]. This may be due to excessively fast transcription rates, leading to uncoordinated mRNA accumulation and translation, and thereby reducing protein synthesis efficiency [135,143]. Thus, selecting promoters with appropriate strength and simultaneously improving translation efficiency is crucial [135,141]. (3) The translation initiation rate of the RBS sequence does not always correlate positively with protein expression level [130,137,140]. For example, RBS206 had a higher predicted translation initiation rate (4,064,405.48 au) than RBS207 (2,311,665.81 au), but its protein expression level (340.12 U/mL) was lower than that of RBS207 (371.87 U/mL) [137], indicating that actual expression efficiency cannot be predicted solely based on the initiation rate [130,137,140]. (4) The secretion mechanism of signal peptides remains unclear [135,150]. Although the N-terminal charge and H-terminal hydrophobicity are known to influence secretion efficiency, simultaneously engineering these parameters does not necessarily improve expression [150]. For instance, SP_Bschi_ (N-terminal charge 4%, H-terminal hydrophobicity 54.5%) achieved over 8000 U/mL, whereas the structurally similar SP_YncM_ (4%, 59.5%) yielded less than 7000 U/mL [150], suggesting more complex regulatory mechanisms in the relationship between signal peptides and secretion efficiency [135,150].

Beyond these individual expression element challenges, interactions between elements significantly impact expression outcomes [139]. For example, the signal peptide can influence not only secretion but also translation initiation at the mRNA level through the interaction between its 5′ region and the short spacer sequence of the RBS, potentially affecting mRNA secondary structure and ribosome binding [139]. In *B. subtilis*, the optimal RBS spacer is typically 7–9 nt [138,145]; however, when paired with the SP_Epr_ signal peptide, an 11 nt spacer increased the expression of cutinase Cut and swollenin EXLX1 by 2.06-fold and 3.23-fold, respectively, whereas a 6 nt spacer only increased expression by 1.09-fold and 1.60-fold (Table 5) [139]. This underscores the importance of synergistic effects between elements [139]. Given the interactions between expression elements described above, sequential optimization often results in a local optimum rather than a global one [106]. The sequential optimization strategy involves individually optimizing each element to its maximum potential and using it as a reference point for subsequent optimization of other elements, thereby facilitating the creation of an optimal expression combination [106]. However, achieving global optimization requires a systematic evaluation of various combinations of different expression components, going beyond mere sequential optimization [106]. This point is clearly illustrated by a study using sfGFP as a reporter: researchers constructed libraries for the promoter (P), RBS, and terminator (T), initially identifying the best individual elements as P_12_, RBS_11E_, and T_8E_ [106]. Subsequently, by constructing a combinatorial library, they identified 33 element combinations exhibiting a 627-fold variation in Relative Fluorescence Intensity (RIF) [106]. Notably, the highest-expressing combination, P_6_-RBS_9_-T_6_, achieved an RIF value of 914.79 × 10^3^, which is 1.95 times higher than that of the sequentially optimized combination P_12_-RBS_11E_-T_8E_ [106]. Furthermore, the interaction between expression elements contributed significantly (53%) to sfGFP expression in *B. subtilis*, compared to only 14% for a gene expressed in *E. coli* [106]. These findings underscore the critical importance of element interactions in *B. subtilis* and highlight the limitations of the commonly used sequential optimization strategy in achieving optimal expression [106]. Therefore, future strategies for optimizing expression elements should not be confined to sequential optimization but must also explore the mechanisms and applications of inter-element interactions [106].

## 5. Conclusions

The optimization research on *B. subtilis* expression vectors has evolved over many years, forming a relatively comprehensive technical system. The systematic framework of “vector skeleton + regulatory systems + expression elements” proposed in this review systematically reviews the existing optimization strategies from an overall perspective. For the vector backbone, the trade-off between stability and copy number is central; while integration vectors offer superior stability, their low copy number often restricts yield. Shuttle vectors like pUC980-1/2 present a promising alternative by achieving high copy numbers and remarkable stability. Regarding regulatory systems, inducible promoters face challenges like leakiness and cost, spurring the development of auto-induction and optogenetic tools. Crucially, the optimization of individual expression elements (promoters, RBS, signal peptides) is complicated by their context-dependent efficiency and significant inter-element interactions. Studies demonstrate that a global optimization approach, which screens combinatorial libraries, can yield far superior results compared to sequential optimization, as element interactions contribute substantially to final expression levels. Thus, future efforts should integrate modular design principles with combinatorial screening to develop versatile, high-performance expression platforms for *B. subtilis*.

## Figures and Tables

**Table 1:** Attributes of common *B. subtilis* expression vectors.

Vector Type	Copy Method	Advantage	Disadvantage	Common Vectors	Reference
Plasmid vector	Self-reproduction	High copy. High expression.	Low stability. Antibiotic dependence.	pEB20, pUB18, pMA5, pHT43, pWB980	[38,42,49,50,51,52]
Integration vector	Integrate into the host genome for replication	High stability.	Low copy. Low expression.	pDL, pDG1662, pAX01, pSG1151, pMutin-GFP, pHT01	[42,53,54]
Thermosensitive phage DNA		High stability. No antibiotic dependence.	Low expression. Less research.	Φ105, Φ105J27, Φ105 dcM	[42,47,48,55]

**Table 2:** *B. subtilis* vector optimization strategies.

Vector	Optimization Process	Conclusion	Reference
pMA5	(1) Integrated expression in *B. subtilis*. (2) Build dual promoters P_HapII_-P_43_. (3) Knockout resistance gene.	(1) Construct a marker-free expression strain for zearalenone-degrading enzyme. (2) Vector stability increased to 98% by the 100th generation, up from 72%.	[56]
BGM	(1) Using plasmid DNA released by lysed *E. coli*. (2) Adopt the Culture mixed method.	(1) Transforming large DNA fragments (>100 kb). (2) Plasmid DNA requires no biochemical purification. (3) Suitable for other hosts. (4) DNA stability remains at 69%.	[55,57]
iREX	(1) Introducing xylite-induced *recA* expression cassette. (2) Deleting the endogenous *recA*.	(1) DNA stability increased from 69% to 73% under xylose induction. (2) DNA stability reaches 93% without xylose induction.	[57]
pl36	(1) Construct plasmids with *spc* and *erm* resistance genes. (2) Use double-resistance screening. (3) Inserted *floB* into recombinant plasmid. (4) Deleting the endogenous gene *floB*.	(1) Couple growth with yield. (2) Yield increased 31.7%. (3) Plasmid loss rate dropped from 34.1% to 11.8%.	[58]
pUBC01	(1) Construct pBV01-incompatible plasmid, pBV02. (2) pBV01 endogenous plasmid ori, pUC-ori, and *kan* genes formed pBV03 plasmid.	(1) Eliminate pBV01. (2) Plasmid stability reached 85% after 40 generations without antibiotic. (3) Efficiently express GFP.	[59]
pHT01	Knock out the *B. subtilis* 168 *yueB* gene.	(1) Enhanced plasmid stability in *BsΔyueB* versus *Bs168*. (2) Acetoin titer increased by 61.99%.	[61]
pHT01	(1) Construct SiteMuB. (2) Knockout genes *yloD*, *yozK*, *yozL*. (3) Enhanced *uvrC* expression. (4) Knockout genes *mfd*. (5) The ChassisLMR-SiteMuB combination.	(1) The mutation rate varied up to 110.63-fold across sites. (2) 41.8% decrease in spontaneous mutation rate. (3) 57.8% decrease in spontaneous mutation rate. (4) 89.1% decrease in spontaneous mutation rate. (5) The stable genetic generation increased 2.1-fold.	[62]
pWB980	(1) Construct pWB980-DB by deleting *bleoR* gene. (2) ori from *E. coli* was inserted into the site upstream of the membrane binding region BA3-1.	(1) Copy numbers increased to 584. (2) Separation stability reached 98%. (3) The alkaline pectinate lyase and the alkaline protease reached 5200 U/mL and 21,537 U/mL.	[63]
pBR322	DNA near the 3′ end of encoding RNA I gene sequence mutation G→T.	(1) RNAI is unable to bind RNAII. (2) Copy numbers increased to 1000.	[64]
pGL3	ColE1 RNA II site-specific mutation C→A.	(1) RNAII promoter strength elevated. (2) RNAII concentration elevated. (3) Increased vector copy number.	[65]
pCB4170	Replication IR III region site-specific mutation C→T.	(1) Initiation protein-replication locus affinity strengthens. (2) Increased vector copy number.	[66]
pDG1730	(1) Targeted integration of P43-DPEase tandem repeats into the *amyE* locus. (2) Knock out the resistance gene *spc*.	(1) Copy number of 3. (2) 2.2-fold increase in DPEase activity. (3) Vector stability declined sharply beyond generations 3–4.	[67]
pJOE8999.1	(1) Knockout of *amyE* generated the BS2 strain. (2) Build dual promoters P_amyQ_-P_cry3A_. (3) P_amyQ_-P_cry3A_-*amyQ* was iteratively integrated into *B. subtilis* BS2. (4) PrsA and SppA overexpression.	(1) Copy number of 6. (2) α-amylase activity increased 20.9-fold. (3) α-amylase production increased to 1439.2 U/mL.	[54]

**Table 3:** Attributes of common *B. subtilis* regulatory systems.

System	Principle	Inducer/Promoter	Features	Reference
IPTG/Lactose	An inducer activates transcription by binding to the repressor protein, which alleviates its suppression of the promoter.	IPTG/Lactose P_grac100_	(1) Widely used. (2) Suffering from induced toxicity and leaky expression.	[78,79,80,81]
Xylose		Xylose/P_xylA/xylB_	(1) Low-cost, readily available inducers. (2) Low utilization rate of inducers. (3) Affected by the CCR effect seriously.	[82,83,84]
Mannitol		Mannitol/P_mtlA_		[85]
Maltose	The inducer combines with the regulatory protein, activating the regulatory protein to transform into a transcription activator, thereby activating transcription.	Maltose/P_glvA/malA_		[86,87]
Methanol		Methanol/P_AOX1_	(1) Low-cost, readily available inducers. (2) Inducer low toxicity.	[88]
Glycerol	Inducer-antiterminator binding disrupts terminator structure, thereby activating transcription.	Glycerol/P_glpD_	(1) Realize self-induced expression. (2) Dependent on medium components. (3) No significant advantage over constitutive expression.	[77]
Fructose		Fructose/P_sacA/sacB_	(1) Inexpensive, non-toxic inducer. (2) Weak promoter and leaky expression.	[89]

**Table 4:** Optimization strategies for the regulatory system in *Bacillus*.

Strain	Optimization Process	Conclusion	Reference
*B. subtilis* 168	Integration of P_xylA_-*araE*-T_fba_ expression box into the *amyE* locus of *B. subtilis* 168.	(1) Strain JY123 was successfully constructed. (2) JY123 fully consumed xylose within 15 h; JY121 (cassette-negative) metabolized only 50% in 19 h. (3) AraE facilitates efficient xylose transport in *B. subtilis*.	[91]
*BSUY00*	(1) Knock out *araR* gene. (2) Expressed *araE* gene. (3) Screen the optimal promoter P_veg_.	(1) 6.25-fold increase in xylose consumption. (2) Fengycin yield of 376.58 mg/L.	[90]
*B. subtilis* J46	(1) Screen galactose-adapted strains *BSGA14*. (2) Mutate the *araR*^H226R^ gene. (3) Knockout of the *glcR* gene involved sugar phosphorylation.	(1) *BSGA14* exhibits enhanced galactose consumption capacity. (2) 2.88-fold upregulation of AraE protein. (3) Protease and β-galactosidase increased by 6.05-fold and 50.31-fold, respectively.	[93]
*B. subtilis* 168	(1) Overexpress the *yjhG* and *yjhH* genes. (2) Knock out *ackA* and *ldh* genes. (3) Introduce an auxiliary pathway composed of *aldA*, *aceB* and *mdh* genes	(1) Improved xylose absorption rate. (2) Fenamycin yield increased by 87%.	[92]
*B. subtilis* 1A751	(1) Truncate the promoter P_malA_. (2) Knock out the maltose hydrolysis gene malL/yvdK.	(1) Significantly increased the activity of promoter P_malA_. (2) Improve the GFP expression. (3) High expression of luciferase and D-aminoacylase. (4) Luciferase and D-aminoacylase expression in this system is superior to that in the constitutive PhapII system.	[95]
*B. subtilis* CCTCCM 2016536	Locus mutations are introduced in the region −2 to +10 of promoter P_amyE_.	The relative activity of D-allulose 3-epimerase was significantly enhanced, reaching 282.43% of the original strain.	[98]
*B. amyliquefaciens* TCCC 19030	(1) Mutant P_amyE_ promoter conserved sites: G3, C8, G9. (2) Break the *cre* sequence symmetry. (3) Adjustment of the relative distance between the *cre* sequence and the transcription start site to 30 bp.	(1) CcpA-CRE binding weakened; downstream gene transcription increased 3.92–5.46-fold. (2) Decrease the CCR effect and increase the P_amyE_ strength. (3) GFP fluorescence intensity increased by 60.87%.	[99]
*B. subtilis* 168	Introduction of A300W, A302W, L306W, and K308W mutations in the CcpA gene.	It cannot exert complete CCR/CCA effects on *xynP* (encode xylose transporter), *ackA*, and *alsS* (encodes the acetyllactate synthase).	[97,100]
*B. amyloliquefaciens* WH1	Knock out the *srfA* gene.	(1) Biofilm formation is thin and fragile. (2) Unable to form spores and carbon metabolism disorder. (3) Downregulation of xylose metabolic gene *xylB* (xylulokinase) and galactose metabolic gene *galK* (galactokinase). High Expression of *ccpA*.	[101]
*B. subtilis* PY79	(1) The light control system CcaSR v0.1 is constructed through the PCB production module, light-sensing module, and transcriptional output module. (2) Optimizing PCB/CcaS expression. (3) Optimizing output promoter activity. (4) Increases dynamic range.	(1) The photoresponsive light control system CcaSR v1.0 was successfully constructed in *B. subtilis*. (2) CcaSR v1.0 achieves more than 70 times activation and fast response dynamics.	[102]
*B. subtilis 168/WB800*	(1) Screen glycerol-specific activation promoters. (2) Regulate the ratio of glucose to glycerol. (3) Overexpression of GlpP.	(1) Successfully developed and constructed an efficient glycerol-induced expression system (GIES) in *B. subtilis*. (2) The efficient expression of aspartate, nattokinase and serine protease was successfully achieved.	[77]

**Table 5:** Optimization strategies for promoter-RBS-signaling peptide-terminator in *B. subtilis*.

Protein	Optimization Process	Multiple/Yield U/mL	Reference
α-amylase AmyZ1	(1) Screen out promoter P_spoVG_.	952.6 (P_spoVG_)	[129]
	(2) Build a double promoter.	1139.8 (P_spoVG_-P_spoVG_)	
	(3) Truncating the 300 bp P_spoVG_ sequence to obtain P_spoVG2_ (180 bp).	1232.3 (P_spoVG_-P_spoVG2_)	
	(4) Introducing a C-to-T substitution in the AT box.	1437.6 (P_spoVG_-P_spoVG142_)	
	(5) Optimization of signal peptide.	1691.1 (Opt3)	
Type I L-asparaginase	(1) Screen out promoter P_yvyD_.	436.28 (P_yvyD_)	[130]
	(2) Build a double promoter.	502.11 (P_aprE_-P_yvyD_)	
	(3) Mutate the -35 and -10 regions of P_yvyD_. within the dual promoters P_aprE_-P_yvyD_.	568.59 (P_aprE_-P_yvyD-Mutant-3_)	
	(4) Screen out RBS10.	790.1 (P_aprE_-P_yvyD-Mutant3_-RBS_10_)	
α-amylase AmyZ1	(1) Screen out double promoter P_veg-ylb_	3687.7 (P_veg-ylb_)	[72]
	(2) Screen out signal peptide SP_NucB_	4199.1 (P_veg-ylb_-SP_NucB_)	
	(3) RBS optimization.	4824.2 (SPNucB-RBS1)	
Aldehyde dehydrogenases	(1) Screen out signal peptide SP_yqzG_.	204.85 (P_aprE_-SP_yqzG_)	[135]
	(2) Screen out promoter P_glv_.	254.82 (P_glv_)	
	(3) Build a double promoter.	268.26 (P_43_-P_glv_)	
L-asparaginase	(1) Screen out promoter P_43_	235.10 (P_43_)	[137]
	(2) RBS sequence optimization.	371.87 (P_43_-RBS_207_)	
Bacilysin	Combine the classic strong SD sequence 5′-TAAGGAGG-3′ with the “ACAAACTC”-8nt interval sequence.	80.3 (*bacA_strongRBS_*)	[138]
GFPmut3	Increase the short-interval sequence from 4 nt to 4–12 nt.	4-fold change (7 nt)	[139]
β-glucuronidase		27-fold change (9 nt)	
Keratinase	(1) Screen signal peptides.	84.3 × 10^3^ (P_43_-SP_dacB_)	[140]
	(2) Mutated RBS sequence.	109.1 × 10^3^ (P_43_-RBS_16D12_-SP_dacB_)	
Thermo-alkaline β-mannanase	(1) Screen signal peptides.	763 (P_hpaII_-SP_lipA_)	[141]
	(2) Screen out promoter P_43_.	908 (P_43_-SP_lipA_)	
Glutaminase	(1) Screening of the optimal signal peptide from 173 candidate signal peptides.	0.24 (P_arpE_-SP_YndA_)	[142]
	(2) Screen out promoter P_HpaII_.	4.52 (P_HpaII_-SP_YndA_)	
	(3) Build a double promoter.	4.90 (P_HpaII_-P_arpE_ -SP_YndA_)	
Hyaluronate lyase	Screening signal peptides increases protein expression.	1.86 × 10^4^ (P_43_-SP_abnA_)	[26]
Alginate lyases	(1) Screen vectors and host bacteria.	0.81 (*B. subtilis* WB600-pP43NMK)	[143]
	(2) Screen out promoter P_nprE_.	4.47 (P_nrpE_)	
	(3) Screen signal peptides.	1.33 (SP_vpr_)	
	(4) Optimize the length and base composition of the RBS short-interval sequence.	4.58 (RBS/NCS-1)	
Upstream GFP/Downstream mCherry	(1) Screening terminators.	Up 2.2-fold change (GFP-TB5)	[144]
		Down 28.4 change (TB5-mCherry)	
	(2) Establishes a double terminator.	Up 2.7-fold change (GFP-TH1.5b-TB5)	
		Down 30.5-fold change (TH1.5b-TB5-mCherry)	

## Data Availability

There is no new data being created.
